# The Association Between Sleep and Quality of Life Among Visitors of Lifestyle Clinics at the Ministry of National Guard-Health Affairs, Western Region, Saudi Arabia

**DOI:** 10.7759/cureus.67087

**Published:** 2024-08-17

**Authors:** Rotan A Baothman, Reem F Alnemari, Saad A Alghamdi

**Affiliations:** 1 Preventive Medicine, Ministry of National Guard Health Affairs, Jeddah, SAU; 2 Preventive Medicine, King Abdullah International Medical Research Center, Jeddah, SAU; 3 Preventive Medicine, King Saud Bin Abdulaziz University for Health Sciences, Jeddah, SAU; 4 Preventive Medicine, King Abdulaziz Medical City, Jeddah, SAU

**Keywords:** sleep hygiene, sleep deprivation, short form-20 qol scale (sf-20), saudi arabia, primary healthcare, pittsburgh sleep quality index (psqi), health promotion

## Abstract

Background: Sleep quality significantly impacts health-related quality of life (QoL). However, the link between sleep quality and QoL needs further exploration, especially in the context of Saudi Arabia.

Objective: To assess the effect of sleep quality on QoL among visitors of lifestyle clinics at the National Guard primary healthcare centers in the Western region of Saudi Arabia in 2023.

Methods: A cross-sectional study was conducted among 369 participants. Data were collected using a questionnaire that addressed sociodemographic characteristics, sleep quality, and perceived QoL. Sleep quality was assessed using the Pittsburgh Sleep Quality Index (PSQI), while QoL was measured using the Short Form-20 (SF-20) QoL scale. Statistical analysis was performed using SPSS (IBM Corp., Armonk, NY).

Results: The median age of participants was 38 years, and the median PSQI score was 9, indicating poor sleep quality. Significant differences in sleep quality were found relating to gender and income. Male gender and low-income status were associated with lower sleep quality. The scores for sleep quality varied between 3 and 16, with a median score of 9, while QoL scores ranged from 34 to 92, with a median score of 72. Good sleep quality was found only among 4.9% of the participants and was associated with higher QoL scores. The correlation between sleep quality and QoL was significant, with a correlation coefficient of -0.399 (p-value < 0.001).

Conclusion: The study identifies a significant correlation between poor sleep quality and lower QoL among visitors of lifestyle clinics in the Western region of Saudi Arabia. These findings suggest the necessity of addressing sleep quality in interventions aimed at improving overall well-being.

## Introduction

Quality of life (QoL) is a subjective concept that reflects an individual's perception of their life position in relation to their aspirations, expectations, standards, and concerns within their cultural context [1]. From the 1980s onwards, the parameters of health-related QoL have broadened to include those elements of overall life quality that have a clear impact on health - be it physical or mental [2]. This concept encompasses both individual mental and physical well-being and the broader community factors that influence population health. These factors include resources, conditions, policies, and practices that shape how people perceive and experience their health.

Many individuals suffer from sleep problems, with numerous distinct sleep disorders identified, insomnia being one of the most common [3]. While some people who feel fatigued during the day have an actual sleep disorder, others simply do not get enough sleep. To function optimally, adults need at least seven to eight hours of sleep per night, yet the average adult gets less than this [3,4]. Sleep hygiene, which includes personal behaviors and environmental routines that promote sleep and avoid activities that disrupt or delay sleep, is crucial [5]. Poor sleep hygiene can lead to sleep-related issues such as insomnia and excessive daytime sleepiness [6-9].

Sleep deprivation, insomnia, and excessive daytime sleepiness are serious public health concerns due to their growing prevalence and potential harmful consequences. These include physical and mental health impairments, decreased productivity, increased accident risk, memory impairment, increased medical utilization, and a higher risk of psychiatric disorders [8,10-12]. Numerous factors such as stressful life events, smoking, caffeine, energy drinks, exposure to light, video games, mobile phone use, as well as unhealthy dietary habits like high carbohydrate glycemic index, saturated fatty acids, deficiencies in omega-3 polyunsaturated fatty acid (PUFA), omega-6 PUFA, amino acids, vitamin D, vitamin C, vitamin B6, and vitamin B12 can all disrupt sleep and exacerbate sleep-related problems [13,14]. Several cross-sectional studies have found a correlation between short sleep duration and lower QoL in the general population [15-17]. However, studies on sleep quality received little attention in Saudi Arabia. Therefore, this study was conducted to assess the association of sleep quality on QoL among visitors of lifestyle clinics at the National Guard primary healthcare centers of the Western region, Saudi Arabia in 2023.

## Materials and methods

Study design

A cross-sectional study was conducted in 2023 at the National Guard primary healthcare centers. The study applied the six pillars of lifestyle medicine, which include a whole-food, plant-based diet, physical activity, restorative sleep, stress management, avoidance of harmful substances, and fostering positive social relationships. These pillars were implemented at the National Guard Health Affairs primary healthcare centers, including specialized polyclinics in Jeddah, Bahra, and Taif.

Participants

The participants were visitors of lifestyle clinics at specialized polyclinics in Jeddah, Bahra, and Taif primary healthcare centers of the National Guard Hospital. The study included all visitors of lifestyle clinics at specialized polyclinics in Jeddah, Bahra, and Taif primary healthcare centers who visited the clinic during the year 2023. However, participants of National Guard healthcare centers who were not referred to lifestyle clinics, individuals aged younger than 18 years old, pregnant women, individuals with morbid obesity (BMI ≥ 40), participants with obstructive sleep apnea, and individuals with two or more multiple comorbidities were excluded from the study.

Sample size and sampling

Based on a conservative anticipated prevalence of sleep-related problems of 50%, a margin of error of 5%, and a confidence level of 95%, we used Epi Info software (Centers for Disease Control and Prevention, Atlanta, GA) to calculate the minimum required sample size. The estimated population of the National Guard primary healthcare centers was approximately 2000, resulting in a minimal calculated sample size of 377 subjects. Participants were allocated using simple random sampling via an Excel sheet (Microsoft Corporation, Redmond, WA).

Data collection methods

Data were collected through a questionnaire addressing sociodemographic characteristics, sleep quality assessment, and perceived QoL. The Pittsburgh Sleep Quality Index (PSQI), a 10-item questionnaire covering subjective sleep quality, sleep latency, duration, efficiency, and disturbances, was employed for sleep quality assessment. Scores ranged from 0 to 21, with higher scores indicating poorer sleep quality [18]. The Short Form-20 (SF-20) QoL scale consists of 20 items measuring six health-related QoL domains. The domains include physical functioning, role functioning, social functioning, mental health, current health perceptions, and pain rate [19]. Where higher SF-20 scores indicate better functioning. Data were collected by qualified, trained residents at the lifestyle clinics after obtaining participants' approval and consent.

Statistical analysis

Statistical analysis was conducted using SPSS (SPSS Statistics for Mac, version 29.0., IBM Corp., Armonk, NY). PSQI and SF-20 were scored to render continuous variables. PSQI was then categorized using a cut-off of 5, where scores higher than 5 indicate poor sleep quality. Continuous variables were tested for normal distribution using Shapiro-Wilk and Kolmogorov-Smirnov tests. As all continuous variables were skewed, we used the minimum, maximum, median, and interquartile range to summarize these variables.

The outcome variable was the total scores of the SF-20, measuring the QoL. Baseline variables were tested for significance with PSQI scores to identify potential confounders using the Mann-Whitney U and Kruskal-Wallis H tests. The association between sleep and QoL was tested using Spearman's rank correlation. Any p-value less than 0.05 was considered statistically significant.

Ethical considerations

Data were securely stored in the principal investigator's office, accessible solely to the authors, ensuring the preservation of confidentiality. Identifying details such as names and identification numbers were deliberately omitted from the data collection form to uphold participant privacy. Ethical approval for the study was duly obtained from King Abdullah International Medical Research Center (protocol number: NRJ23J/132/05). In addition, the study ensured that participation was entirely voluntary, with informed consent obtained from all participants. Participants were informed about the purpose and the nature of the study.

## Results

A total of 378 participants were initially included in the study. After applying the exclusion criteria, nine participants were removed, resulting in a final sample of 369 participants. The ages of the participants ranged from 18 to 83 years, with a median age of 38 years and an interquartile range (IQR) of 28 to 55 years. The total scores for sleep quality varied between 3 and 16, with a median score of 9 and an IQR of 7 to 11.

Of the total participants, 35.8% were males (n = 132) and 64.2% were females (n = 237). The participants were distributed across various marital statuses with a majority being married (53.7%, n = 198). Income levels varied across the participants with 27.6% earning 5,000 or less. Significant differences in sleep quality were found in relation to gender and income. Male participants had a lower mean rank for PSQI total scores (P = 0.001). As for income, participants earning 5,000 Saudi riyals (SAR) or less had the highest mean PSQI rank, suggesting lower sleep quality among this income group (p = 0.001). Table 1 provides a comprehensive overview of the distribution of demographic characteristics and their association with sleep quality among the study participants.

**Table 1:** Distribution of demographic characteristics and their association with sleep quality (PSQI mean rank) among the study participants (n = 369). * P-value calculated using the Mann-Whitney test. ** P-value calculated using the Kruskal-Wallis test. PSQI: Pittsburgh Sleep Quality Index.

Variable		Groups	N	%	PSQI mean rank	P-value
Gender	Male		132	35.8%	161.5	0.001*
	Female		237	64.2%	198.1	
Marital status	Single		123	33.3%	190.9	0.163
	Married		198	53.7%	175.1	
	Divorced		39	10.6%	213.9	
	Widow		9	2.4%	197.1	
Income	5,000 or less		102	27.6%	218.0	0.001**
	5,001-10,000		83	22.5%	157.5	
	10,001-12,000		38	10.3%	175.3	
	12,001 or more		146	39.6%	180.1	
Body mass index	Underweight		18	4.9%	144.6	0.409
	Normal		129	35.0%	184.7	
	Overweight		141	38.2%	187.5	
	Obese		81	22.0%	190.1	

The study observed various lifestyle factors and their association with sleep quality. No significant association was found between sleep quality and smoking status. Smokers represented 21.4% of the total participants. Those who consumed caffeine in both the morning and evening made up 45.5% of the participants, but the differences across the groups regarding sleep quality were not statistically significant. The participants' meal preparation habits varied, with 33.6% cooking by themselves, 50.9% eating from home, and 15.4% eating from a restaurant. All lifestyle factors did not reveal any statistical differences in sleep quality. Table 2 displays statistics and associations between lifestyle factors and sleep quality.

**Table 2:** Descriptive statistics and associations between lifestyle factors and sleep quality among the study participants (n = 369). PSQI: Pittsburgh Sleep Quality Index.

Variable	Groups	N	%	PSQI mean rank	P-value
Smoking	Yes	79	21.4%	195.1	0.339
	No	290	78.6%	182.3	
Caffeine intake	Morning	105	28.5%	163.7	0.096
	Evening	60	16.3%	186.1	
	Morning and evening	168	45.5%	195.6	
	None	36	9.8%	196.0	
Preparing meals	I cook by myself	124	33.6%	184.5	0.102
	Eat from home	188	50.9%	177.3	
	Eat from restaurant	57	15.4%	211.5	
Videogames (per day)	No	237	64.2%	178.5	0.271
	One hour or less	64	17.3%	194.0	
	More than one hour	68	18.4%	199.3	
Phone time (per day)	Three hours or less	116	31.4%	179.5	0.496
	More than three hours	253	68.6%	187.5	

The scores for physical functioning ranged from 6 to 18, with a median score of 17. Role limitations had scores ranging from 3 to 6, with a median of 4. Mental health scores ranged from 9 to 26, with a median of 19. Health perception scores were varied, with a minimum score of 7, a maximum score of 25, and a median score of 18. Social functioning scores ranged from 1 to 6, with a median score of 5. Pain scores ranged from 1 to 6, with a median score of 4. The total scores for QoL ranged from 34.07 to 92.31, with a median score of 72.53 and an IQR of 64 to 80. Detailed results for the SF-20 scores among the study participants are presented in Table 3.

**Table 3:** Descriptive statistics of Short Form-20 content scores among the study participants.

Component	Minimum	25 percentile	Median	75 percentile	Maximum
Physical	6.00	14.00	17.00	18.00	18.00
Role	3.00	4.00	4.00	4.00	6.00
Mental	9.00	16.00	19.00	22.00	26.00
Perception	7.00	15.00	18.00	21.00	25.00
Social	1.00	4.00	5.00	6.00	6.00
Pain	1.00	3.00	4.00	5.00	6.00

**Figure 1:**
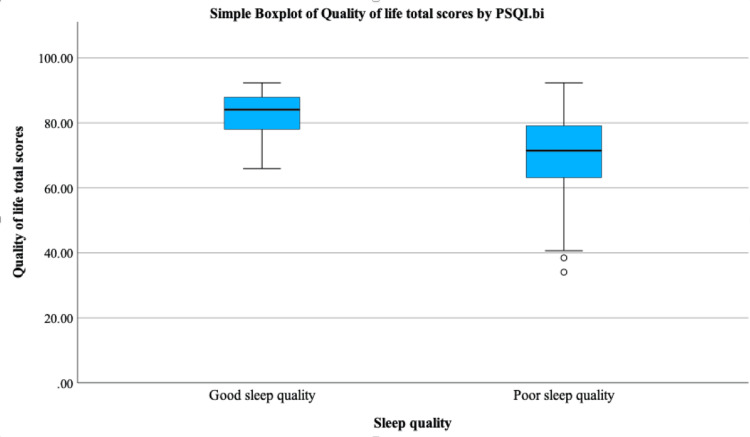
Boxplot illustrating the association between sleep quality and quality of life. P-value < 0.001, calculated using the Mann-Whitney test. PSQI: Pittsburgh Sleep Quality Index.

The correlation between sleep quality and QoL was found to be significant. The correlation coefficient was -0.399, indicating a moderate negative relationship between sleep quality and QoL. This suggests that as sleep quality improves (i.e., lower PSQI scores), the QoL also improves (p < 0.001). Good sleep quality was found only among 4.9% of the participants. For participants with good sleep quality, the total QoL scores spanned from 66 to 92, with a median of 84 and IQR from 78 to 88. In contrast, participants with poor sleep quality had total QoL scores ranging from 34 to 92, with a median of 71 and IQR from 63 to 79. Overall, this figure suggests a potential association between sleep quality and QoL, with good sleep quality potentially linked to higher QoL. Figure 1 demonstrates the association between quality of sleep represented by PSQI categories and QoL represented by SF-20.

## Discussion

Sleep has a significant impact on a person's physical and mental health as well as their productivity and level of energy, all of which contribute to a healthy lifestyle. The current study found a moderate association between better sleep and a higher QoL. Similarly, a recent longitudinal study supports these findings, demonstrating that both extending sleep duration and improving sleep quality can enhance QoL [20]. In addition, a cross-sectional study conducted in Australia found a link between a shorter sleep duration and a lower standard of living [21]. A survey carried out in the primary healthcare centers (PHCCs) in southwestern Saudi Arabia revealed that very few people with insomnia practice sleep hygiene, and that daytime sleepiness is indicative of an unhealthy lifestyle that might have a substantial impact on sleep cycles [22].

The duration and quality of sleep can significantly be influenced by various demographic factors of participants. Gender plays a significant part in it. In contrast to our findings that men have lower-quality sleep, a cross-sectional study conducted in Spain among the general population found that women had considerably lower sleep quality than men [23]. The explanation might be that women frequently report higher stress levels associated with balancing social, familial, and professional obligations. This stress can result in lower-quality sleep. Sleep patterns in females can be impacted by hormonal shifts during the menstrual cycle, pregnancy, and menopause. Hormonal changes, for instance, might cause sleep disorders such as insomnia, restless legs syndrome, or dyspnea. In contrast, higher levels of physical activity, which are often reported in males, can promote better sleep quality. Males may have different stress responses compared to females, potentially impacting sleep. Effective stress management can contribute to better sleep quality. A cross-sectional study of adults conducted in southwest Ethiopia revealed a statistically significant correlation between participants' monthly income and their quality of sleep [24]. The current study also showed that those with lower incomes typically have worse quality sleep than people with higher incomes. Concerns about providing for basic needs, employment insecurity, and unstable finances could all be contributing factors. Work schedules can be influenced by income levels. Higher earners frequently have more control over their work schedules, whereas lower earners may work irregular or longer hours, work nights, or have numerous jobs, all of which might interfere with their sleep cycles. Poorer health outcomes associated with low-income status, such as chronic illnesses, pain, and mental health disorders, can contribute to sleep disturbances [25]. Limited access to nutritious food can lead to poor dietary habits, which in turn may affect sleep quality. Studies conducted worldwide, including one in China, have revealed that married individuals sleep better than single or widowed ones [26]. However, the present research found no such association. In the current study, body mass index did not correlate with the quality of sleep, which runs contradictory to numerous previous studies. For example, a survey conducted on university students in Zagreb indicated that a high body mass index and obesity could be contributing factors to bad sleep [27].

People's sleep habits have been significantly impacted by leading healthy lifestyles. These practices include working out on a regular basis, abstaining from drugs and tobacco, maintaining a healthy diet, and setting screen time limits. Smokers had poorer sleep quality, according to a cross-sectional analysis performed on individuals from the CoLaus-HypnoLaus research [28]. Similarly, another Australian study discovered that adults' sleep quality can be negatively impacted by coffee consumption. In reference to screen time, a Saudi Arabian study claims that using a phone before bed can result in both poor sleep quality and afternoon drowsiness [29]. A different study found a correlation between playing video games intensely and having poor-quality sleep [30]. However, our study found no correlation between sleep quality and smoking, caffeine consumption, mobile phone usage, and video games.

The center-based design of the study, which could not accurately represent the general community, is one of the study's limitations. It leaves out people who are not referred to lifestyle clinics, therefore it might not include people with various sleep patterns or aspects of their QoL. The generalizability of results to these populations, who frequently face unique sleep issues, may be limited if people with particular health concerns (such as morbid obesity or obstructive sleep apnea) are excluded. The accuracy of reported sleep quality and QoL is impacted by recall bias and social desirability bias in data gathered through patient interviews and self-reported questionnaires (e.g., PSQI and SF-20). The study found disparities in sleep quality between genders and income levels, although it might not have fully included other demographic characteristics. (e.g., education level and ethnicity) that could influence sleep outcomes. While the study focused on the six pillars of lifestyle medicine, other relevant factors impacting sleep quality (e.g., sleep environment and access to mental health services) were not comprehensively explored.

## Conclusions

Our study findings demonstrated a moderate, significant association between sleep quality and QoL among visitors of lifestyle clinics at the National Guard primary healthcare centers in the Western region of Saudi Arabia. The majority of our participants reported poor sleep quality, underscoring the prevalence of sleep disturbances in this population. Importantly, individuals with better sleep quality exhibited higher QoL scores, suggesting a potential causal link. To effectively enhance QoL, interventions targeting sleep improvement should consider factors such as gender and income, as our study revealed significant differences in sleep quality across these demographics. Further research is warranted to elucidate the underlying mechanisms of this relationship and to develop tailored interventions to improve sleep quality and, consequently, overall well-being.
